# Clinical relevance of routine transvaginal ultrasound in women referred with pelvic organ prolapse

**DOI:** 10.1186/s12905-021-01173-z

**Published:** 2021-01-13

**Authors:** Lone Pedersen, Marianne Glavind-Kristensen, Pinar Bor

**Affiliations:** 1 Department of Obstetrics and Gynaecology, Randers Regional Hospital, Randers, Denmark; 2 Department of Obstetrics and Gynaecology, Aarhus University Hospital, Aarhus, Denmark; 3 Department of Clinical Medicine, Aarhus University, Aarhus, Denmark; 4 Moellehatten 21, 4. 1, 8240 Risskov, Denmark

**Keywords:** Pelvic organ prolapse, Ultrasonography, Incidental finding

## Abstract

**Background:**

The aim of this study was to investigate the prevalence of incidental findings on transvaginal ultrasound scan in women referred with pelvic organ prolapse by a general practitioner and to investigate which further examinations and treatments were performed as a result of these findings.

**Methods:**

This was a retrospective cohort study that investigated women with pelvic organ prolapse referred to the outpatient urogynaecological clinics at Randers Regional Hospital and Aarhus University Hospital, Denmark.

**Results:**

A total of 521 women were included and all of them were examined with a routine transvaginal ultrasound scan and a gynaecological examination. Prolapse symptoms only and no specific indication for transvaginal ultrasound scan were seen in 507 women (97.3%), while 14 women (2.7%) received scans on indication. Among the latter women, five (35.7%) had cancer. In the women with solely prolapse symptoms, 59 (11.6%) had incidental findings on transvaginal ultrasound scan, but all were benign. However, two patients were later diagnosed with cancer unrelated to the initial ultrasound findings. The treatment was extended with further examinations not related to POP in 19 of the women (32.2%) with incidental ultrasound findings.

**Conclusion:**

The prevalence of incidental ultrasound findings was not high in the women referred with pelvic organ prolapse and no additional symptoms, and all these findings were benign. However, it should be considered that these findings resulted in further investigations and changes to the patients’ initial treatment plans. A meticulous anamnesis and digital vaginal examination are crucial to rule out the need for vaginal ultrasound.

## Background

Pelvic organ prolapse (POP) is a herniation of the pelvic organs into the vagina and includes cystocele, rectocele, enterocele, vaginal vault prolapse and uterine prolapse. It is diagnosed during gynaecological examinations in 41–50% of women above the age of 50 years [1, 2]. Only about 6% of these women have classic prolapse symptoms such as a feeling of bulging, a heavy feeling, protrusion, pressure or a visible bulge into the vagina [3]. POP is also associated with functional pelvic floor disorders such as urinary or faecal incontinence, bladder emptying problems and urinary tract infections. These symptoms sometimes motivate women to see a gynaecologist due to the negative effect on their quality of life [3].

In Denmark, a transvaginal ultrasound scan (TVS) of the uterus and ovaries is usually performed as a supplement to a gynaecological examination to investigate whether the patient can empty the bladder properly or to see if there are any utero-ovarian pathologies as these can cause some of the symptoms presented by the patient (e.g. heaviness or findings of a pelvic mass). Moreover, these pathologies may influence the choice of treatment for the primary POP [4]. It is important to note that the choice of treatment for prolapse is primarily based on women’s subjective complaints and not on ultrasound results or objective examinations. However, some women referred with POP symptoms also have unspecific symptoms such as unintended weight loss, uncharacteristic pelvic pain, postmenopausal vaginal bleeding or anaemia, which could be related to malignant conditions [5].

There are no current guidelines about routine ultrasound screening in asymptomatic women, but several studies have suggested that routine ultrasound is a good examination to perform before POP surgery to determine if there are any pre-malignant or malignant pathologies of the uterus, ovaries or cervix [6–8]. Other studies have found endometrial screening [9, 10] and screening of the ovaries [11, 12] with ultrasound in asymptomatic women to be ineffective in differentiating benign findings from malignant findings. Notwithstanding, there still seems to be a tendency to use routine TVS preoperatively for all women undergoing gynaecological examinations [13, 14].

To the best of our knowledge, no previous study has exclusively investigated the incidental ultrasound findings, such as ovarian cysts, fibromas, endometrial thickening or polyps in the uterus, in women with POP. These conditions are often asymptomatic; however, women may be subject to comprehensive additional investigations, including different invasive procedures, biopsies, blood tests and even operations, which may place them at risk of complications such as infections, reoperations and bleeding. Furthermore, false-positive screening tests are associated with increased levels of worry that continues for many years [12].

The aim of this study was to investigate the prevalence of incidental findings detected by routine TVS, including uterine, cervical and ovarian pathologies, in women referred with POP. Moreover, the study aimed to investigate which further examinations and treatments these women undergo due to these incidental findings, along with the related consequences, complications and results. Accordingly, the clinical relevance of routine TVS in women referred with POP was evaluated.

## Methods

This retrospective cohort study investigated all the women with POP referred to the outpatient gynaecological clinics at Randers Regional Hospital and Aarhus University Hospital by a general practitioner in 2015. Clinical information including age, previous hysterectomies, previous cancers, type of pelvic organ prolapse, the results of the ultrasound, the treatment of the prolapse and further investigations, controls and complications due to the incidental findings of TVS were recorded from electronic patient records.

**Fig. 1:**
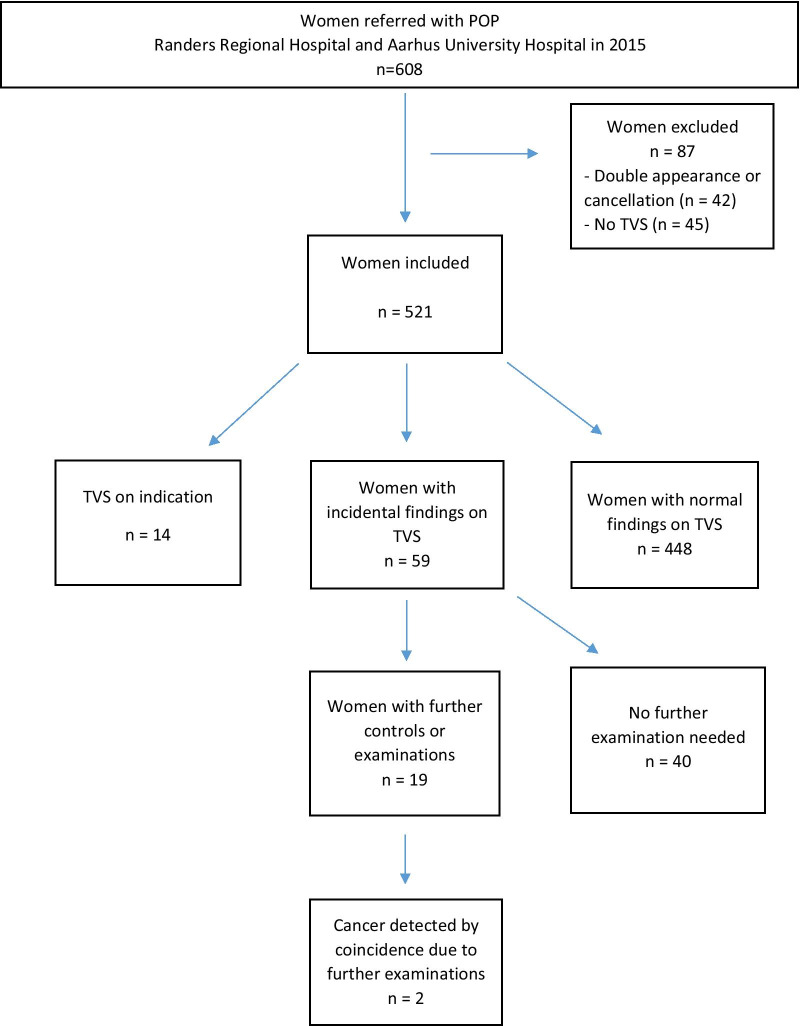
Flowchart of incidental findings on TVS in women referred to the outpatient clinics with POP. POP, pelvic organ proplase; TVS, transvaginal ultrasound

We excluded women referred with POP but never examined with TVS (Fig. 1). Furthermore, patients with a double appearance in the two departments and women who cancelled their appointments were excluded.

A diagnosis of POP is based on a gynaecological examination to determine the type of prolapse, which is mainly divided into the following three categories: anterior compartment (cystocele), medial compartment (uterine prolapse, vaginal vault prolapse) and posterior compartment (enterocele and rectocele). The Baden–Walker classification system was used to grade the POP. The system consists of four grades: grade 0—no prolapse, grade 1—halfway to hymen, grade 2—to hymen, grade 3—halfway past hymen and grade 4—maximum descent.

This was a descriptive study, so statistical tests between subgroups were not relevant. The characteristic variables were summarised and calculated as percentages and mean (SD).

## Results

A total of 608 women were referred with POP to the two urogynaecological clinics in 2015, but only 566 of these women were examined at the clinics (Fig. 1). In 45 of these women (8%), ultrasound examination was not performed. Thus, 521 women were included in the study (Table 1).

**Table 1:** Demographics of patients referred with POP

Characteristics	
Age (mean, SD)	63 (0.63)
	n (%)
TVS on indication TVS without indication	14 (2.7%) 507 (97.3%)
Previous hysterectomy No previous hysterectomy	96 (18.4%) 425 (81.6%)
Prolapse diagnosis*	
Anterior compartment Posterior compartment Central compartment Several compartments No prolapse	207 (39.7%) 79 (15.2%) 32 (6.1%) 167 (32.1%) 36 (6.9%)
*Previous cancer diagnosis*	
Yes No	31 (5.9%) 490 (94.1%)

A total of 5.9% of the women (n = 31) had a previous cancer diagnosis, including breast cancer (n = 18), lymphoma/leukaemia (n = 4), brain cancer (n = 2), malignant melanoma (n = 1), bladder cancer (n = 1), thyroid cancer (n = 1), cervix cancer (n = 2), endometrial cancer (n = 1) and ovarian cancer (n = 1).

Of the 507 women with solely POP symptoms, 448 (88.4%) had normal ultrasound examinations. Incidental findings detected by TVS were seen in 59 women (11.6%) (Table 2). Further examinations were performed in 19 women (3.7%, correlating to 32.2% of the women with incidental findings) because of these incidental findings, but all were benign.

**Table 2:** Ultrasound findings and further treatment of patients referred with POP

	n (%)
*TVS without indication*	
Normal Incidental findings Endometrial pathology Ovarian pathology Fibromas	448 (88.4%) 59 (11.6%) 9 (1.8%) 15 (2.9%) 35 (6.9%)
*TVS on indication*	
No cancer diagnosis Cancer diagnosis	9 (64.3%) 5 (35.7%)
*Further investigations of incidental findings*	
Ovarian pathology Blood test (Ca-125), laparoscopy, control TVS/gynaecological examination (9/15) Bilateral salpingo-oophorectomy (2/9) Fibromas Hysterectomy (2/35) Endometrial pathology Abrasions, mini-hysteroscopy, biopsies (8/9) Hysteroscopic polyp removal (3/8)	**15 (2.9%)** 9 (60%) 2 (13%) **35 (6.9%)** 2 (5.7%) **9 (1.8%)** 8 (89%) 3 (33%)
*Indication for operative treatment*	
POP Incidental findings + POP Incidental findings	285 (54.7%) 9 (1.7%) 8 (1.5%)
No indication for operative treatment	219 (42.1%)
*More than one visit to the clinic due to incidental findings on TVS*	
Yes No	10 (16.9%) 49 (83.1%)
*Complications due to incidental findings on TVS*	
Yes No	0 (0%) 59 (100%)

TVS, transvaginal ultrasound scan; POP, pelvic organ prolapse

Table 2 presents the ultrasound findings. Ovarian cysts varied from 2 to 6 cm were found in 15 women (2.9%). Nine of these women (60%) were examined further with blood tests (including the tumour marker CA-125), laparoscopy or additional controls. Both laparoscopic oophorectomy and POP surgery were performed in two of these women (13%). Uterine fibromas were found in 35 women (6.9%), and two of these women (5.7%) had vaginal hysterectomy and cystocele repair due to POP. Finally, nine women (1.8%) had endometrial pathology, which resulted in further examinations with abrasions, mini-hysteroscopy and biopsies in eight (89%) of these women. Benign polyps were found and removed surgically in three (33%) of these women. We did not find any cervical pathologies in the women included in this study. In two of the women (0.39%) without alarm symptoms, cancer was detected incidentally without relation to the ultrasound examination; one of the women had endometrial cancer, detected by a final histological examination of the uterus after the POP surgery. She had a history of breast cancer. The other women had endometrial cancer detected by an additional TVS for the control of a benign ovarian cyst.

In 14 women (2.7%), there was a strong indication for ultrasound examination due to alarm symptoms of malignancy, a pelvic mass detected during the gynaecological examination or a different reason, such as intrauterine device control, and five of these women (35.7%) received a cancer diagnosis (Table 2).

A total of 10 (16.9%) of the women with incidental findings on TVS had more than one visit to the clinic due to incidental findings on TVS. None of the women examined further for incidental findings had complications. A total of 285 women (54.7%) underwent only POP surgery, while nine of the women (1.7%) had an additional operation together with the POP surgery due to incidental findings. Eight women (1.5%) had surgery due to the incidental findings only, without POP surgery (Table 2). There was no indication that any operation was performed for 219 women (42%) referred with POP.

## Discussion

Our study on women referred to an outpatient clinic with a diagnosis of POP showed that 92% had a routine TVS examination in addition to a gynaecological examination. Most of these women (97.3%) only had prolapse symptoms, and among these women, 11.6% had incidental findings that were benign. However, these incidental findings detected by TVS influenced the choice of treatment or led to further examinations in 32.2% of these women. There were strong indications for ultrasound examination due to malignancy alarm symptoms in only 14 women (2.7%), and five of these women were diagnosed with cancer (35.7%).

The use of TVS in women with POP is claimed to be a routine screening procedure, which means the examination of women without any symptoms. POP can be accompanied by a variety of symptoms, like the sensation of a bulge in the vagina, heaviness in the lower abdomen and functional disorders, as described by *Rortveit* et al. [3]. The best correlation has been found between the sensation of a bulge in the vagina and objective prolapse beneath the hymen, whereas other symptoms have not been well correlated to the objective findings of POP, as explained by *Gutmann* et al. [15]. Gynaecological examination, including vaginal exploration for enlargement of the pelvic organs, is often supplemented with TVS in Denmark since this modality can reveal structural changes in the pelvic organs and may therefore explain women’s symptoms. In our study, 6.9% of the women referred with POP had subjective symptoms but no objective findings of POP. TVS should be considered a diagnostic tool to rule out other explanations of unspecific symptoms and should not be classified as a screening procedure in women with suspected POP, whereas a good anamnesis and a digital vaginal examination is considered of great importance.

Our findings indicated that the use of TVS in asymptomatic women with POP is not beneficial in detecting malignancy since the prevalence of malignancy in this patient population is generally low. Some of the asymptomatic women (3.7%) in our study underwent further investigations because of benign incidental findings, and only a few asymptomatic women were actually diagnosed with cancer (0.39%). However, in the two women, cancer was not detected using the primary TVS, but was detected by final histological examination of the uterus after POP surgery and by an additional TVS for the control of a benign ovarian cyst.

On the other hand, the use of ultrasound was more helpful in women for whom gynaecological examination revealed a pelvic mass or women with early warning symptoms because 35.7% of the women (n = 5) in our study who had indications that warranted ultrasound (n = 14) actually had cancer. As seen in our results, women with a history of cancer had a higher frequency of malignancy than other women with POP; 3.2% (one out of 31 patients) with a previous cancer had a new cancer diagnosis compared to 0.21% (one out of 476 patients) of the asymptomatic women who had never had cancer. Even though our material was limited, it seems that the risk of urogenital cancer is around 15 times higher if a patient has had cancer before, which may be an argument in favour of routine TVS in this specific group of patients.

The ultrasound scans in our study missed at least two endometrial cancers, and this is consistent with the literature of *Havrilesky* et al. [13], which claimed that ultrasound has a sensitivity of 97% and a specificity of 55% for the detection of endometrial cancer with an endometrial stripe greater than 5 mm. This means that 3% of women with cancer or precancerous conditions of the endometrium are missed with TVS, while 45% of women have a risk of undergoing further investigations even though they are not sick. The studies of both *Ramm* et al. [9] and *Wan* et al. [10] found that TVS for asymptomatic women is unreliable when it comes to the detection of cancer. They respectively found a 0.7% prevalence of cancer after biopsy and/or ultrasound and an overall incidence of pre-malignant and malignant changes in the uterus in 0.94% of asymptomatic women without ultrasound prior to surgery. This is consistent with our findings of cancers detected coincidentally after the primary POP surgery (0.39%).

Previous studies by *Frick* et al. [6] and *Renganathan* et al. [8] on women with POP, which included 681 and 517 postmenopausal women, respectively, described the low prevalence of unanticipated endometrial cancer in 0.3% and 0.8% of the women, respectively. These findings are comparable to those of our study. Another study by *Grigoriadis* et al. [7] found a 0% prevalence of endometrial cancer where the women were examined by TVS prior to surgery, yet 2.7% of the women in the study population had unanticipated pre-malignant or malignant uterine pathologies based on biopsies. All these studies suggest that routine preoperative ultrasound can reduce the risk of unexpected pathologies in postmenopausal women even though the prevalence is low.

In our study, 2.9% of the asymptomatic women were diagnosed with a pathology of the ovaries via TVS, and 60% of these women had to undergo further investigations to qualify the ultrasound findings. Ultimately, no malignant ovarian pathologies were found. *Partridge* et al. [11] found that ultrasound often fails to differentiate benign from malignant ovarian tumours and that TVS screening is associated with a low positive predictive value, which means that women undergo unnecessary surgery as a consequence of TVS findings. *Reade* et al. [12] similarly recognised that TVS is a poor screening procedure for ovarian cancer in asymptomatic women.

The subjective symptoms of women with POP are important and an indicator of treatment, it is not the objective findings of prolapse or TVS that determine whether a woman should be offered treatment. However, we found that incidental findings on TVS sometimes change the primary POP treatment plan. For instance, 33% of the women in our study with thick endometrial lining on TVS actually had polyps removed at the time of their primary POP surgery, and 13% of the women with ovarian cysts had an oophorectomy as well as their primary POP surgery. Furthermore, 5.7% of the women with fibromas had a hysterectomy in addition to POP surgery. This means that a total of 13.5% of the women had a different or additional surgical treatment because of benign incidental findings. Seen from the perspective of the patient, it is reassuring to know that the uterus and ovaries are normal when some symptoms are present. In this study, 58% of the women had surgery because of their POP symptoms or incidental findings. Interestingly, 42% of the women who were referred with POP never had an operation. Some of these women were treated conservatively, but some merely went to the doctor to make sure that there was nothing malignant, and the TVS helped provide them with the assurance they needed. On the other hand, the women who had benign incidental findings on TVS underwent further investigations and had to face the fear of having a malignant condition, which, according to *Reade* et al. [12], is associated with considerable psychological consequences.

Two details worth noting from this study are the low prevalence of patients readmitted to hospital and the absence of complications after surgery for incidental findings. Generally, the investigations of the incidental findings were not as comprehensive as expected, and this limited how much physical damage was incurred. However, a questionnaire sent to all the women would have provided us with a more realistic picture of the real complications and the psychological conditions of these women, which would have been interesting to evaluate.

To the best of our knowledge, the frequency of incidental findings on TVS in a population of women with POP and the consequences for these women have not previously been investigated. Although POP is a common condition and a valid reason for gynaecological examinations in women, we still do not have any proper knowledge about this issue.

The strength of this study was the comprehensive experience of all gynaecologists in Denmark with TVS, where it is a routine form of examination in almost all women attending clinics. This means that TVS is easy and accessible for use by most gynaecologists, and the study conditions correlate well with practice. Additionally, TVS is not a very expensive procedure, and in Denmark, there is no need to refer patients to specialist clinics to obtain TVS, which was a major advantage in this study.

Nevertheless, this study had some limitations. We could have differentiated between postmenopausal and premenopausal women to see if the prevalence of incidental findings was higher among postmenopausal women, as *Frick* et al. and *Renganathan* et al. [6, 8] suggested. We could also have looked at factors such as height, weight, familial risk of cancer and smoking status, which may be correlated with malignancy. However, due to the small number of patients in this study, it was not possible to undertake these subgroup analyses. Another action we could have taken to improve the validity of our results would have been to match our patients with age-appropriate controls without any symptoms to compare the effects of screening in patients with POP.

## Conclusion

The prevalence of incidental ultrasound findings in women referred with POP and no additional symptoms was not high, and the findings were often benign. It should be considered that these incidental findings result in further investigations and changes to the initial treatment. A meticulous anamnesis and a good clinical examination, including a digital vaginal examination, are crucial before deciding if TVS can be omitted. Our study suggests that in women with solely prolapse-related symptoms and objective findings of POP, TVS could be omitted from routine examinations. However, more research on a large population is needed to make an evidence-based conclusion regarding the necessity of routine TVS in this population.

## Data Availability

A part of the data generated and analysed during this study are included in this published article and its supplementary information files. The datasets generated and analyzed during the current study are not publicly available due to Danish legislation in this area. Thus, consent for publication is “Not Applicable”. Data are available from the corresponding author on reasonable request.

## Abbreviations

TVS: Transvaginal ultrasound scan
POP: Pelvic organ prolapse
